# Investigation of nanoscale structural alterations of cell nucleus as an early sign of cancer

**DOI:** 10.1186/2046-1682-7-1

**Published:** 2014-02-10

**Authors:** Yang Liu, Shikhar Uttam, Sergey Alexandrov, Rajan K Bista

**Affiliations:** 1 Biomedical Optical Imaging Laboratory, Department of Medicine, Department of Bioengineering, University of Pittsburgh, Pittsburgh, Pennsylvania, USA; 2 Tissue Optics & Microcirculation Imaging Group, School of Physics, National University of Ireland, Galway, Ireland; 3 Hillman Cancer Center, UPCI Research Pavilion 2.32, 5117 Centre Avenue, Pittsburgh, Pennsylvania 15232, USA

## Abstract

**Background:**

The cell and tissue structural properties assessed with a conventional bright-field light microscope play a key role in cancer diagnosis, but they sometimes have limited accuracy in detecting early-stage cancers or predicting future risk of cancer progression for individual patients (i.e., prognosis) if no frank cancer is found. The recent development in optical microscopy techniques now permit the nanoscale structural imaging and quantitative structural analysis of tissue and cells, which offers a new opportunity to investigate the structural properties of cell and tissue below 200 – 250 nm as an early sign of carcinogenesis, prior to the presence of microscale morphological abnormalities. Identification of nanoscale structural signatures is significant for earlier and more accurate cancer detection and prognosis.

**Results:**

Our group has recently developed two simple spectral-domain optical microscopy techniques for assessing 3D nanoscale structural alterations – spectral-encoding of spatial frequency microscopy and spatial-domain low-coherence quantitative phase microscopy. These two techniques use the scattered light from biological cells and tissue and share a common experimental approach of assessing the Fourier space by various wavelengths to quantify the 3D structural information of the scattering object at the nanoscale sensitivity with a simple reflectance-mode light microscopy setup without the need for high-NA optics. This review paper discusses the physical principles and validation of these two techniques to interrogate nanoscale structural properties, as well as the use of these methods to probe nanoscale nuclear architectural alterations during carcinogenesis in cancer cell lines and well-annotated human tissue during carcinogenesis.

**Conclusions:**

The analysis of nanoscale structural characteristics has shown promise in detecting cancer before the microscopically visible changes become evident and proof-of-concept studies have shown its feasibility as an earlier or more sensitive marker for cancer detection or diagnosis. Further biophysical investigation of specific 3D nanoscale structural characteristics in carcinogenesis, especially with well-annotated human cells and tissue, is much needed in cancer research.

## Background

Cancer develops through a series of genetic and epigenetic events that ultimately result in structural changes in the cell nucleus. As such, the structural abnormality of the cell nucleus (also known as nuclear morphology) is one of the hallmarks in cancer and remains the gold standard for cancer diagnosis and prognosis. Due to the diffraction-limited resolution (~250-500 nm) of conventional light microscopy, the characteristic morphological changes identified in cancerous or precancerous cells are limited to mostly micron-scale features, such as increased nuclear size, irregular nuclear shape and coarse chromatin texture. Many structural abnormalities observable at the micro-scale do not occur until an advanced stage, making it difficult to distinguish early-stage cancers from benign conditions. Further, in the era of personalized medicine, the detection of pre-cancer or early-stage cancer is not sufficient. As many pre-cancers or early-stage cancers will never progress into invasive cancer, such detection in fact may lead to unnecessary treatment in the absence of aggressive cancer that does more harm than good to the patient at a high cost. Therefore, it is crucial to not only identify pre-cancer or early-stage cancer, but also predict which pre-cancer or early-stage cancer is likely to develop into a more invasive form (i.e., prognosis). The conventional microscale nuclear morphology has some prognostic value, but its accuracy is somewhat limited in many clinical scenarios.

On the other hand, the nanoscale structural properties, also referred to as “nano-morphology”, show the potential to become a new class of morphological markers for earlier and more accurate cancer diagnosis and prognosis. It is well recognized that cancer is a complex disease involving early changes in the genome and epigenome [1]. The nucleus, as the storehouse of the genomic information, is not a homogeneous organelle with randomly organized DNA; instead, the DNAs are packed at various densities and spatially arranged in a certain manner in a 3D space that is associated with nuclear function [2-4]. Recent studies using super-resolution microscopy also confirm that histone octamers are not randomly distributed throughout the nucleus and that pronounced differences are seen in the compaction of chromatin with such fluctuation in histone density [5]. The spatial organization of specific chromatin domains with a size in the hundred nanometer range also plays an essential role for gene regulation [4]. During carcinogenesis, the 3D spatial arrangement of chromatin patterns experience translocation and alterations in the spatial density of chromatin at different loci of the nucleus. For example, the large-scale changes in 3D genomic architecture or the changes in spatial distribution of chromosome have been reported in cancer [6,7]. Therefore, we hypothesize that the complex genomic and epigenomic changes in carcinogenesis result in nanoscale structural alterations arising from the changes in the 3D spatial arrangement and the chromatin density variation in the cell nucleus. In other words, investigating the nano-morphology characteristics as the downstream structural manifestation of complex genetic and epigenetic events regardless of which molecular pathways are involved in carcinogenesis is an important effort. As such physical characteristics can be detected easily with low-cost, high throughput and high sensitivity, yet independent of molecular heterogeneity, they have the potential to become a new class of cancer markers to make a significant clinical impact. For example, the analysis of cellular disorder strength has been reported to detect nano-architectural changes early in carcinogenesis that precede microscopically detectable cytological abnormalities [8] and show the ability to detect cancer from normal cells from a remote location in lung, colon and pancreas [9-11].

Elucidation of 3D nano-morphological changes in cancer requires tools that are able to interrogate nano-structures in the cell nucleus and other sub-cellular components. Thanks to the remarkable advances in optical microscopy techniques in recent years, the advanced light microscope now supports the detection of structural properties at a scale about an order of magnitude less than what conventional light microscope can detect. The nanoscale structural characteristics can be measured either using direct imaging with nanoscale resolution, or indirect analysis of optical signals from light-cell interaction. The direct nanoscopic imaging can be achieved by super-resolution fluorescence microscopy techniques, such as stochastic optical reconstruction microscopy (STORM) [12], photoactivatable localization microscopy (PALM) [13], stimulated emission depletion microscopy (STED) [14] and 3D structured illumination microscopy (3D-SIM) [15]. These super-resolution microscopy techniques have the ability to directly image the nanoscale architecture of any labeled molecular component in live and fixed cells and even tissue at a spatial resolution down to 20–50 nm. The label-free nanoscopic imaging of biological cells at a resolution of 90 nm based on optical diffraction tomography has also been reported recently [16]. A few investigators have pioneered the investigation of 3D nanoscopic imaging of nuclear architecture in cancer cells [5,17]. As the super-resolution fluorescence microscopy becomes commercially available, it will play an essential role in elucidating how nanoscale structural arrangement in the nucleus is altered during carcinogenesis. While these are powerful basic research tools, the clinical translation of these techniques has several challenges to overcome. Due to the complex fluorescent staining process, high cost, sophisticated instrument operation and the very low throughput, they are not well suited as a routine clinical diagnostic tool at the current form.

An alternative to direct nanoscopic imaging is indirect measurement of nanoscale structural properties by probing optical properties via scattered light. Although it often does not directly visualize the structures at nanoscale resolution [as is the case of super-resolution imaging], the structural properties from a single cell or sub-cellular organelle can be indirectly quantified with nanoscale sensitivity and accuracy by light-scattering based microscopy techniques. Light scattering is an intrinsic optical signal caused by the spatial variation of intra-cellular macromolecular densities. This approach has the advantages of simple sample preparation (no molecular staining or labeling required), simple instrument and operation, low cost, high throughput and high sensitivity. Such advantages are crucial in translating these techniques into a real clinical setting to improve cancer diagnosis and prognosis.

A wide variety of light-scattering microscopy techniques have been developed to analyze the nano-morphology characteristics, and can be generalized into the following four categories based on the underlying physical principles to achieve nanoscale structural assessment. Please note that these are broad categories that are not mutually exclusive, and in fact, can and do have overlap. The first form is to quantify the cell structure by measuring phase difference using interferometry-based approach, such as various versions of quantitative phase microscopy and digital holographic microscopy (DHM) [18]. The light interference effect is well known to detect changes in optical path length even at sub-nanometer sensitivity. The second form is based on the analysis of Fourier space (or **K**-space, the conjugate of an object) of scattered light, such as optical scatter imaging [19] and spectral-encoding of spatial frequency (SESF) [20-22]. This approach is based on the concept that any scattering object’s structure can be described either by the spatial distribution of refractive index or its Fourier components in the far-field [23]. Although the image resolution is still limited by diffraction, the 2D or 3D structural characteristics of a scattering object can be quantified with a nanoscale precision by directly assessing the spatial frequencies in **K**-space. The third form is to combine microscopic imaging with light scattering spectroscopy. In this case, the scattered light is collected as a function of scattering wavelength or/and angle. Assuming the scattering object as a spherical or spheroid shape, the structural parameters such as size distribution from a well-defined microscopic region are derived from a model-based interpretation (e.g., Mie-theory, T-matrix). For example, in confocal light absorption and scattering spectroscopic microscopy [24], the smallest scatterer is about 100 nm [25]. The fourth form is referred to as partial-wave spectroscopy that characterizes the statistical properties of refractive index fluctuation by disorder strength [8] which is proportional to the amplitude and the length scale of the macromolecular density variations at the scale of ~20 nm.

In this paper, we will focus on reviewing the use of two spectral-domain optical microscopy techniques – spatial-domain low-coherence quantitative phase microscopy (SL-QPM) and spectral-encoding of spatial frequency (SESF) microscopy – from the first two-categories to interrogate the nanoscale structural information. First, we review the general theory behind SL-QPM and SESF microscopy and why the nanoscale structural alterations can be interrogated. Next, we discuss the technical implementation and how these techniques are designed to analyze the human cell and tissue samples. In the third section, we present the results of how nanoscale nuclear architecture is altered during carcinogenesis in cancer cell lines and well-annotated human tissue at different stages of cancer.

## Methods

The SL-QPM and SESF microscopy are two optical microscopy systems that are able to assess the 3D nanoscale structural information with just simple reflection-mode optical microscopy setup, without high-NA immersion objectives or demanding nano-positioning mechanical scanning, well-suited for low-cost and high throughput clinical use. They share a common theory of light scattering by an inhomogeneous scattering object and first Born approximation [23]. It does not assume any a priori information about the scattering object (e.g., shape, size) and the only assumption is that the scattering object is a weakly scattering object with relative refractive index between the scattering object and surrounding medium is close to 1, which can be broadly applied to live or fixed biological cells and tissue with easy sample preparation. Both methods also share a common experimental approach of assessing the Fourier space (i.e., **K**-space) by various wavelengths to quantify the structural information of the scattering object, which can be easily implemented with tunable light source or spectral device. Further, both techniques are capable of assessing 3D nanoscale structural information without the need for axial scanning.

### General theory of light scattering by an inhomogeneous object

Consider a scalar plane wave incident on the scattering object described by

(1) $$E_{i}\left(r,k,t\right)=A\left(k\right)e^{i\left(k_{i}r-\mathrm{\omega t}\right)}$$

**Figure 1:**
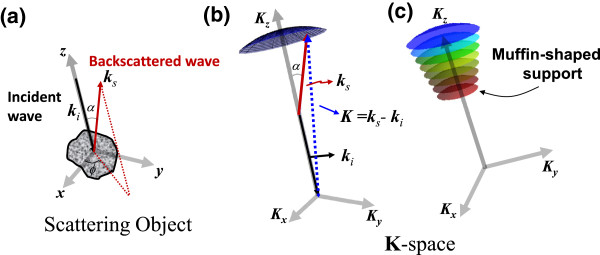
**Representation of the scattering vectors in K-space in the reflection configuration. (a)** Illustration of the light interaction with the scattering object, where *α* is the backscattering angle, and *Φ* is the azimuthal angle. **(b)** Representation of the scattering vector for a single wavelength in **K**-space (spatial-frequency space), where ***k***_i_ is the incident wave vector and ***k***_s_ is the scattered wave vector. **(c)** Representation of scattering vectors in K-space with different wavelengths (from 400 to 700 nm).

The 3D structure of the scattering object can be described using 3D scattering potential [23] as

(2) $$F\left(r\right)=k^{2}\left(n^{2}\left(r\right)-1\right)/\left(4\pi \right)$$

where *k* is the wavenumber and *n* is the free-space refractive index and **r** is the location within the scattering object. Assuming that the plane-wave is incident at an illumination angle θ (θ **=** 0, as shown in Figure 1a) and under the first Born approximation [23] (Figure 1b) which applies to weakly scattering object (i.e., relative refractive index between the scattering object and surrounding medium is close to 1), the scattering amplitude in the far field is expressed as

(3) $$f_{s}\left(k_{i},k_{s}\right)=\int_{V\left(r'\right)}F\left(r'\right)e^{i\left(k_{s}-k_{i}\right)\cdot r'}d^{3}r'$$

The Fourier transform of the scattering potential in **K**-space is expressed by

(4) $$\tilde{F}\left(K\right)=\int F\left(r'\right)e^{-iK\cdot r'}d^{3}r'$$

where the **K**-space vector is defined as

(5) $$K=k_{s}-k_{i}$$

Thus, the scattering amplitude in Eq. (3) turns into

(6) $$f_{s}\left(k_{i},k_{s}\right)=\tilde{F}\left(K\right)=\tilde{F}\left(k\left(s-s_{0}\right)\right)$$

Therefore, the scattering amplitude of the scattering object under first Born approximation depends only on the vector **K** defined in Eq. (5), or Fourier components of the scattering potential [23,26].

The Fourier components of the 3D scattering potential in **K**-space (or spatial frequency space) can be represented as an Ewald’s sphere [23]. The light collection numerical aperture (NA) limits the accessible Fourier components or spatial frequency ranges of the scattering object, which are represented as the Ewald’s sphere cap (Figure 1b). When multiple wavelengths are used, a succession of Ewald’s sphere caps is obtained that define a muffin-shaped region of spatial frequency support in **K**-space for reflection geometry (Figure 1c). Every point on the Ewald’s sphere corresponds to a specific 3D spatial-frequency vector:

(7) $$K=k_{x}x+k_{y}y+k_{z}z$$

where

(8) $$k_{x}=2\mathrm{\pi n}\left(\sin\alpha -\sin\theta \right)\cos\phi /\lambda$$

(9) $$k_{y}=2\mathrm{\pi n}\left(\sin\alpha -\sin\theta \right)\sin\phi /\lambda$$

(or *k*_*x*_ and *k*_*y*_ can be collectively expressed as the lateral spatial frequency

(10) $$k_{r}=2\mathrm{\pi n}\left(\sin\alpha -\sin\theta \right)/\lambda$$

and

(11) $$k_{z}=2\mathrm{\pi n}\left(\cos\theta +\cos\alpha \right)/\lambda$$

(*θ*: incident angle; *α*: scattering angle; *φ*: azimuthal angle; *λ*: wavelength, *n*: refractive index). These equations can alternatively be expressed by the corresponding spatial periods defined as

(12) $$H_{x}=\frac{2\pi }{k_{x}},H_{y}=\frac{2\pi }{k_{y}},H_{z}=\frac{2\pi }{k_{z}}$$

If the complex amplitudes of all of the possible 3D Fourier components (or spatial frequencies) are collected, the 3D scattering object (scattering potential) can be reconstructed via 3D inverse Fourier transform, which serves as the basis for optical diffraction tomography [23,27]. However, the 3D structure in the reconstructed scattering object still has the diffraction-limited resolution due to the NA-limited accessible bandwidth of Fourier components and integration process of Fourier transform. A recent advance overcame this limitation by expanding the collected spatial frequencies of the scattering object using a high-NA oil immersion objective together with a deconvolution algorithm to achieve a resolution of 90 nm [16] in the reconstructed 3D scattering object.

Alternatively, based on the above theory, the nanoscale structural characteristics of the 3D scattering object can also be interrogated via two simple light microscopy systems using a moderate NA (NA ≤ 0.5): Fourier-filtering based SESF and a spectral interferometry based SL-QPM. These approaches do not directly image the scattering object at the nanoscale resolution, but quantify the nanoscale structural information by employing the spectral information of scattered light from the 3D scattering object. These approaches can be implemented with simple optical setup and are widely applicable to live or fixed biological cells, as well as clinically prepared fixed tissue.

### Spectral-encoding of Spatial Frequency (SESF)

#### General description of SESF

Spectral-encoding of spatial frequency (SESF) is a simple way to quantify the nanoscale structure of a 3D scattering object by encoding each spectral wavelength with a structure-characterizing axial spatial frequency [20,21]. According to the Fourier theory of image formation, the structural components of a complex object can be quantified by a distribution of spatial frequencies in the Fourier space, describing its various spatial scales from large (i.e., low spatial frequency) to small (i.e., high spatial frequency). Thus, each pixel in the microscopic image is represented by a distribution of spectral colors characteristic of the corresponding axial structure at that image point. The 3D axial structure at each pixel can thus be quantified at nanoscale accuracy with a simple reflection-configuration optical microscope setup. It also provides a unique structure-color based image contrast and real-time structural characterization.

**Figure 2:**
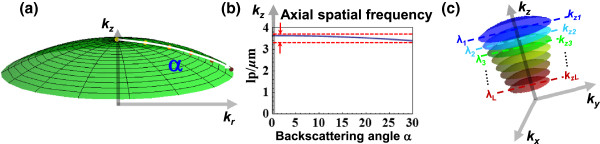
**The correspondence between axial spatial frequency and wavelength in the reflection configuration. (a)** Ewald’s sphere cap at a normal incidence in the reflection configuration. **(b)** The axial spatial frequency (*k*_*z*_) is only weakly dependent on the backscattering angles or lateral spatial frequency (*k*_*r*_). **(c)** Each axial spatial frequency (*k*_*z*_) can be encoded by each wavelength with a small uncertainty as indicated by two red dashed lines on Figure **(b)**.

The theoretical basis of SESF can be easily understood in the context of Ewald’s sphere. As shown in the Ewald’s sphere cap (Figure 2a), we note that, for a reflection configuration with a normal illumination angle (*θ* =0°) and moderate light collection NA (e.g., NA ≤ 0.5), the spatial frequency along the axial (z) direction *k*_z_ (or spatial period H_z_) at a given wavelength is only weakly dependent on the backscattering angles or *k*_r_ (Figure 2b). We can assume each axial spatial frequency of the image point can be encoded with a given wavelength, regardless of what is the backscattering angle within a moderate collection NA, as illustrated in Figure 2c. By measuring the intensity distribution as a function of wavelength for each pixel of the microscopic image directly on the image plane using a spectral device, the axial spatial frequency (or spatial-period) profile of a 3D structure at each image point can be reconstructed with Eq. (11). Alternatively, the dominant axial structure (one dominant value of spatial period) at each image point can be visualized as a corresponding color in real time.

Furthermore, if the complex amplitude of the backscattered light in the Fourier space can be collected, when applying the SESF principle, the simultaneous reconstruction of 3D tomographic image and the quantitative characterization of 3D structural information at the nanoscale accuracy from any volume of interest within the 3D object can also be realized [22].

#### Nanoscale accuracy

A key advantage of SESF is its nanoscale accuracy in determining the axial spatial period of the structure at each image point. As discussed above, each axial spatial frequency of the image point can be encoded with a given wavelength with a small uncertainty. The theoretical uncertainty for determining the axial spatial period (Δ*H*_*z*_) can be estimated by the following equation

(13) $$\Delta H_{z}=\lambda \left(1-\cos\alpha \right)/n\left(1+\cos\theta \right)\left(\cos\theta +\cos\alpha \right)$$

For example, for a normal incidence (θ = 0°) and the light collection NA of 0.5 (α = 30°), the estimated uncertainty for axial spatial period is Δ*H*_*z*_ = 20 nm with the corresponding maximum possible error of ±10 nm. A smaller NA results in a smaller error, however, there is an inherent trade-off between the image resolution and the accuracy in axial structural characterization.

**Figure 3:**
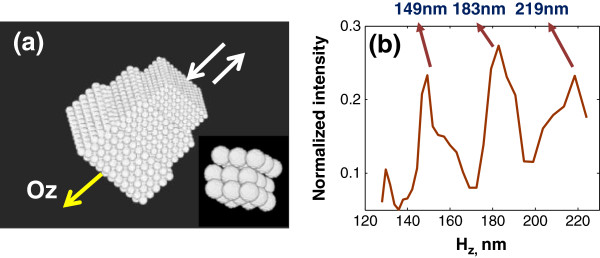
**Numerical validation of SESF approach: (a)** The structure of the simulated 3D object, illuminated along Oz axis; the insert is a magnified portion of the object. **(b)** The reconstructed axial spatial-period profile averaged over the entire image area.

A numerical experiment was performed to validate the nanoscale accuracy of SESF approach. A 3D model object was constructed, composed of three known mixed-sized nanosphere aggregates. Each nanosphere aggregate unit consists of 10 by 10 by 10 nanospheres with diameter d (*d* =240 nm, 200 nm and 150 nm, respectively) (Figure 3a). The spatial period of each nanosphere aggregate unit is defined by [*d* sin(*π*/3) + 10] nm in the numerical model, corresponding to the axial spatial period of 218, 183 and 140 nm, respectively. A plane wave with spectral range of 400–700 nm at Δλ =10 nm is used. The backscattered waves for each wavelength, within the collected NA (NA = 0.5), are generated with the Born approximation [23]. In the averaged axial spatial-period profile (Figure 3b), the peak of three nano-structural units (219, 183 and 149 nm) are within the theoretical error of ±10 nm compared to the actual spatial period in the constructed numerical model (218, 183 and 140 nm), which confirms the theoretical nanoscale accuracy of ±10 nm at NA = 0.5.

#### Experimental setup

**Figure 4:**
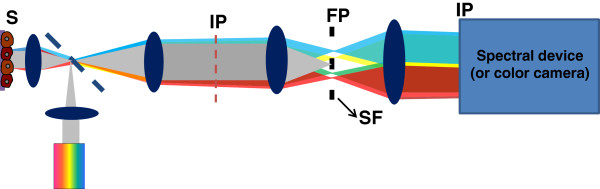
**The schematic of SESF system.** S: sample; IP: image plane; FP: Fourier plane; SF: Spatial filter.

The schematic of the SESF system is shown in Figure 4. This setup is built upon a commercial microscope frame (AXIO Observer, Carl Zeiss) using reflection configuration at normal illumination. A broadband white-light source (Xenon-arc lamp 150 W, Newport Inc) was collimated and the backscattered light was collected by the objective (NA = 0.5). The annular-shaped spatial filter (SF) was used on Fourier plane (FP) to collect the spectral signals for all accessible scattering and azimuthal angles simultaneously. This SF suppresses the zero-order signal and removes the contribution of non-informative zero-order broadband spectrum from each image point, to provide the required bandwidth of spatial frequencies and spectral range for spectral encoding of the axial spatial frequency. On the other hand, the annular Fourier mask also effectively uses the NA of the optical system to form a relatively high resolution image. The SESF and bright-field images were recorded using either color CCD camera (AxioCam HRc, Carl Zeiss) on the image plane. Alternatively, a spectral device such as spectrometer (Acton Research) is also used to obtain the spectroscopic data on the image plane.

#### Experimental demonstration using a model system

**Figure 5:**
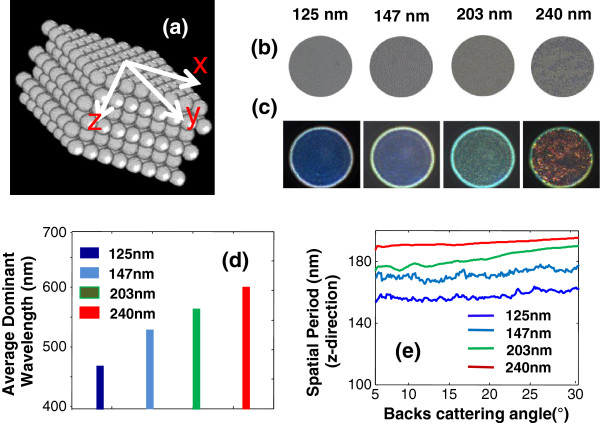
**Real-time quantitative structural imaging of nanosphere aggregates using SESF. (a)** Illustration of the nanosphere aggregate configuration. At the image plane of the SESF system, **(b)** bright-field and **(c)** SESF images of nanosphere aggregates with four different sizes: 125 nm, 147 nm, 203 nm and 240 nm. **(d)** The average axial spatial period for the dominant structure of each sample. **(e)** Distribution of axial spatial period on the backscattering angle for each sample.

The ability of SESF system for real-time quantitative structural imaging of a complex 3D object and nanoscale sensitivity was demonstrated using nanosphere aggregates of known sizes: 125 ± 3 nm; 147 ± 3 nm; 203 ± 5 nm; 240 ± 5 nm, as illustrated in Figure 5a [21]. The nanosphere aggregate is formed by a self-assembling process [21,28,29]. The optical thickness of the nanosphere aggregate was ~30 μm, suggesting a multi-layer structure. Figures 5B-C show the bright-field and corresponding real-time SESF images of nanospheres captured with a colored CCD camera. The nanosphere size is well beyond the lateral resolution limit of our optical system, indistinguishable in conventional bright-field images, but the *axial* structural information can pass through the optical system using SESF. The nanoscale size differences are clearly presented as distinct colors in the SESF images. The dominant color in each SESF image is directly correlated with the dominant spatial frequency of the axial structure, which depends on nanosphere size. It should be noted that what we measure with SESF is the axial spatial period, which is not always equivalent to the size of the nanosphere aggregate. As size increases, the axial spatial period increases and the SESF image shows progressively red-shifted color as predicted by Eq. (11). The average dominant wavelengths (Figure 5d) also show a progressive increase. Even a 22 nm difference can be distinguished by spectral color shift, suggesting a nanoscale sensitivity in detecting structural changes.

### Depth-resolved spatial-domain low-coherence quantitative phase microscopy (SL-QPM)

#### Theoretical basis

Along with assessing the nanoscale structural information in **K**-space, the 3D nanoscale structure of a scattering object can also be quantified using the quantitative phase information derived from a modified spectral interferometry approach. As discussed above, the scattered wave from an inhomogeous weakly scattered object can be depicted by the Ewald’s sphere [23,30], and the 3D spatial frequency, after being scattered from the scattering object, is given by **K** = **k**_**s**_ – **k**_**i**_.

Compared to other spectral-domain interferometry setups, the unique aspect of the SL-QPM is that it takes advantage of the small refractive index mismatch between the sample and the mounting medium in the clinically prepared biological sample and implicit coherent gating inherent in the spectral interferometry to detect the sub-resolution internal structural change in a depth-resolved manner, rather than the sample thickness difference (further discussion below).

**Figure 6:**
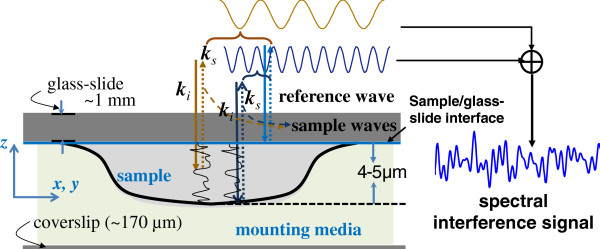
**The configuration of glass-slide based cell or tissue sample.** The reflected wave at the glass-sample interface serves as a reference wave, and the backscattered wave due to the structural heterogeneity inside the cell serves as a sample wave. The reference wave can also enhanced by putting a reflection coating at the sample/glass slide interface. The mounting medium is placed between the sample and the coverslip.

The SL-QPM uses the standard configuration of clinically prepared cell or tissue slides to create an interferometry configuration. As shown in Figure 6, for normal illumination and the collection along the axial *z*-direction in the reflection configuration, the backscattered waves are restricted to the axial *z*-direction, and the spatial frequency reduces to **k**_**z**_ = 2*k***z**, where **z** is the unit vector in the positive *z*-direction in **K**-space. The backscattered wave from the sample described by Eq. (6) is superimposed with the reference wave reflected at the glass-sample interface, resulting in an interference signal expressed by [31]

(14) $$P\left(k\right)=S\left(k\right)\left[r_{r}^{2}+\int_{0}^{Z}r_{s}^{2}\left(z^{'}\right)\mathrm{dz}+2\int_{0}^{Z}r_{s}\left(z^{'}\right)r_{r}\cos\left(2\mathrm{kn}\left(z^{'}\right)z^{'}\right)dz^{'}\right]$$

where *S*(*k*) is the power spectrum of the source, *r*_*r*_ is the reflection coefficient of the reference wave, *r*_*s*_(*z*) is the scattering coefficient of the sample at depth *z*, *Z* is the total sample thickness and *n*(*z*) is the refractive index distribution along the axial *z*-direction. The Fourier inverse of Eq. (14) results in the following equation:

(15) $$\begin{array}{ll} p\left(z_{\mathrm{opl}}\right) & =2\Gamma ⊗\left[\left(R_{r}+R_{s}\right)\delta \left(0\right)+2r_{r}{ℱ}^{-1}\right)\\ & \;\times \left(\left(\int_{0}^{Z}r_{s}\left(z^{'}\right)\cos\left(2\mathrm{kn}\left(z^{'}\right)z^{'}\right)dz^{'}\right)\right]\left(z_{\mathrm{opl}}\right) \end{array}$$

(where $R_{r}=r_{r}^{2}$ and $R_{s}=\int_{0}^{Z}r_{s}^{2}\left(z^{'}\right)\mathrm{dz}$) which is a convolution of the source correlation function Γ with the superposition of the reference wave and the backscattered sample wave. This mathematical relation suggests that the source correlation function serves as an implicit coherent window that separates the phase information at each optical depth whose resolution is limited by the coherence length. The amplitude of the Fourier-transformed signal at any given optical depth of interest gives the optical path length (OPL) distribution along the axial direction of the sample, while the phase at each fixed optical depth of interest captures the sub-resolution nanoscale change in OPL at that location. This sub-resolution *change* in OPL is calculated based on Eq. (15),

(16) δpzopl=λ02πarctanImpzoplRepzopl

where *z*_opl_ is the fixed optical depth location, Im and Re denote the imaginary and real parts of the complex convolution *p*(*z*_*opl*_) respectively, and *δp*(*z*_opl_) is the optical path length difference (OPD) at a specific optical depth location *z*_opl_, which is not limited by the resolution of optical system and can be used to probe the nanoscale structural changes of the biological cell.

#### SL-QPM instrument

**Figure 7:**
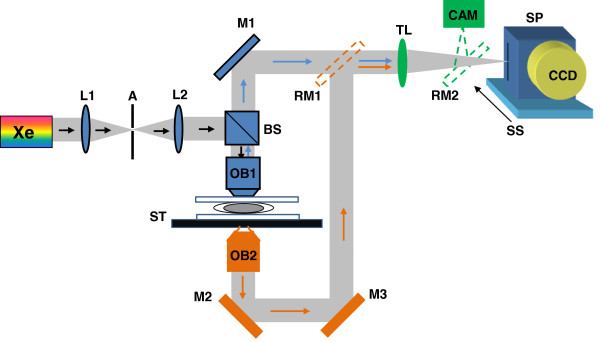
**The schematic of SL-QPM setup.** Xe: Xenon lamp; L: lens; OB: objective; M: mirror; RM: removable mirror; TL: tube lens; SP: spectrograph; SS: scanning stage.

We developed a simple optical microscopy system using low-coherence white light, referred to as spatial-domain low-coherence quantitative phase microscopy (SL-QPM), to implement the above-discussed approach. As shown in Figure 7, the SL-QPM is that based on a reflection configuration, and the inherent configuration of clinically prepared biological samples (shown in Figure 6) creates the common-path interferometry configuration. Such configuration helps eliminating the phase noise in the conventional interferometry configuration, in which the reference and sample waves have separate optical paths. The use of a broadband white light from Xenon-arc lamp of low-coherence light source helps eliminating the speckle noise. The reflectance image from the sample was collected by a scanning imaging spectrograph (Acton Research, MA) and a CCD camera (Andor Technology, CT) that recorded a three-dimensional spatial-spectral intensity cube *I*(*x*, *y*, *k*), which is then transformed into an OPD map *δp*(*x*, *y*, *z*_*opl*_) at a specific *z*_*opl*_ based on Eqs. (15-16).

#### Nanoscale sensitivity

The structural sensitivity, which is defined as the ability of this system to detect the smallest OPD change, is not limited by the resolution of light microscopy, but limited by the system stability. We investigated the temporal stability of the depth-resolved values at the fixed optical depth locations of 1.5 μm, 3 μm, 4.5 μm, and 6 μm, respectively. The depth-resolved values are relatively stable with a mean standard deviation around 1 nm due to temporal fluctuations, which is 0.76 nm, 0.78 nm, 0.86 nm and 1.2 nm at the optical depth locations of 1.5 μm, 3 μm, 4.5 μm, and 6 μm respectively. This standard deviation of mean OPD value of ~1 nm determines the nanoscale sensitivity. Please note that the nanoscale sensitivity is *not* nanoscale resolution. It does not directly image an object at the nanoscale resolution, but detect the structural *changes* at the sensitivity of ~ 1 nm. So the experimental interpretation of OPD value is only meaningful in the context of comparing the relative structural changes.

#### Analysis of SL-QPM internal nanoscale structural changes

Our goal is to use SL-QPM to probe the nanoscale structural changes associated with the biological processes. However, in most spectral interferometry setup, the nanoscale change in OPL comes from the structural changes at a distinct physical interface with a strong refractive-index mismatch. In most cell or tissue preparation, the biological cells have a strongest refractive index mismatch at the interface of cell and mounting medium. As a result, such measurement often reflects the surface variation of the cell rather than the internal structural changes. The cell surface variation is sometimes non-specific and subject to the cell preparation artifact.

To probe the nanoscale changes in the internal structure of the cell or tissue, we prepared the biological samples with a small refractive index mismatch between the sample and the mounting medium. Thus the sample wave predominately comes from backscattered waves from inside the scattering sample, not the reflection from the sample-mounting medium interface.

**Figure 8:**
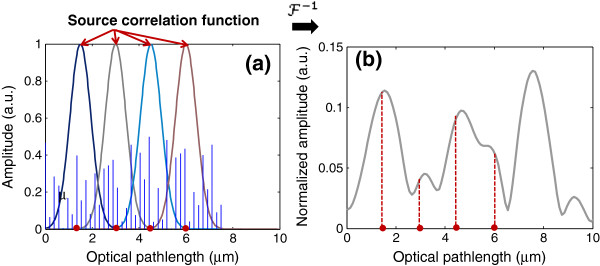
**Illustration of depth-resolved SL-QPM via coherence gating at multiple fixed depth locations (shown as red dots). (a)** The illustration of convolution of source correlation function and the original profile of the scattering object (blue stem-plot). **(b)** The derived OPL profile from SL-QPM system.

Another important property of the SL-QPM system is its implicit coherent gating inherent in the spectral interferometry. According to Eq. (15), the Fourier-transformation of the spectral interference signal is a convolution of the source correlation function Γ (i.e., the power spectral density of the source) and the actual OPL profile of the scattering object. As an example, we assume the true OPL profile of the scattering object as the distinct blue stem-plot in Figure 8a. Due to the limited spectral bandwidth of the light source, the source correlation function, as indicated in Figure 8a, serves as a window for coherent gating to restrict the detected OPD to come from the back-scattered waves within this window around the given optical-depth of interest, without being affected by the scattered waves from those optical depths at one or more coherence-length apart. The derived OPL profile from the SL-QPM system is illustrated in Figure 8b, where the original OPL profile from the scattering object is modified by the source correlation function. If multiple fixed optical depth locations are chosen such that the distance between them is at least one coherence length apart [indicated by the red dots in Figure 8a], we can capture the internal structural changes within the coherence-gated optical section around each optical depth. The physical conditions for this approach to be effective include a closely matched refractive index between the sample and mounting medium and the signal strength at the selected locations is above the noise floor.

#### Validation of SL-QPM to probe internal nanoscale structural changes

**Figure 9:**
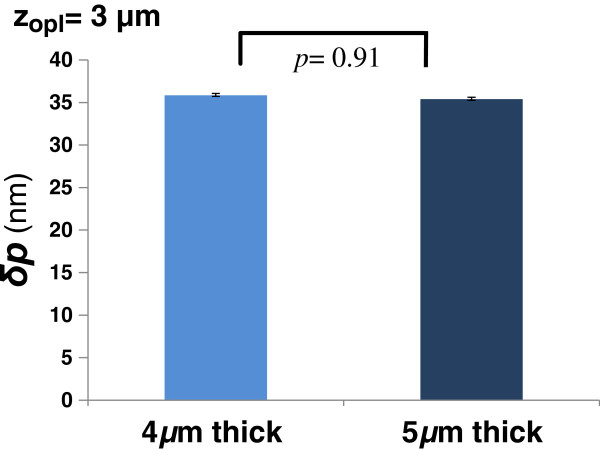
**The OPD (δp) for 4 μm and 5 μm thick sections at fixed optical-depth locations at the optical depth of 3 μm.** The OPD remains the same in the similar tissue area, independent of tissue thickness. The error bar represents the standard error based on the analysis of approximately 40 cells from the similar area of two serial sections.

We conducted experiments to validate that SL-QPM indeed probes the internal structural changes, rather than the non-specific surface variation due to the sample preparation artifact [31]. Our experiment is based on the hypothesis that if we use the same tissue sample that is sectioned at the different thickness, the implicit coherence gating should ensure that the detected OPD from an internal depth location is the same, independent of the section thickness. We used serial tissue sections of the small intestinal tissue from a normal mouse sectioned at 4 *μ*m and 5 *μ*m using a microtome, placed on the coated glass slide, coverslipped with a mounting medium (n = 1.50), without any staining. As the tissue sections are serial sections of the same tissue segment, it is reasonable to assume that these two tissue sections have similar structural properties, but different thickness. With the SL-QPM instrument and the method discussed above, we analyzed the spectral interference signal from a similar tissue area – expressed as a function of spatial frequency **K** and then extracted *δp*(*x*, *y*, *z*_*opl*_) at the optical depth inside the sample (*z*_*opl*_ =3 μm), as shown in Figure 9, clearly supporting that the OPD indeed detect the internal structural changes, rather than the variation in sample thickness.

## Results and discussion

In this section, we will show some examples of using SESF and SL-QPM based methods to detect nanoscale structural changes in a fundamental biological process important in cancer, as well as to demonstrate the ability to detect pre-cancerous changes in clinical samples beyond what conventional light microscopy can detect.

### Nanoscale structural changes in abnormal cell growth

**Figure 10:**
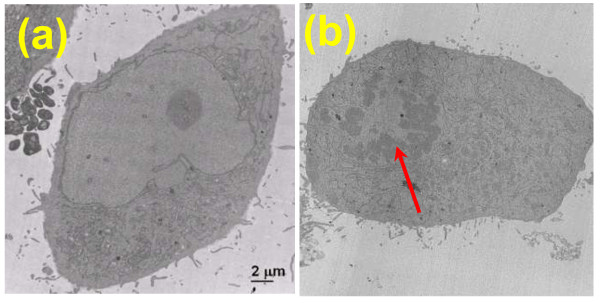
**The transmission electron microscopy (TEM) images of cells arrested at (a) G**_**1**_**/S phase and (b) G**_**2**_**/M phase, respectively.** The red arrow indicates the presence of large high-order chromatin clusters in the nucleus of cells at G_2_/M phase.

As cancer is a result of uncontrolled cell growth, understanding the nanoscale structural changes in the abnormal cell growth is an important first step in their use to detect pre-cancerous changes. We performed a proof-of-concept study to analyze the nanoscale structural changes in the nuclei of HeLa cells during abnormal mitosis, one of the hallmarks in cancer. We use an established cancer cell line, HeLa cell, arrested at two distinct phases of cell cycle – G_1_/S and G_2_/M, as described previously [32]. We used a drug – thymidine – that inhibits DNA synthesis to arrest cells at G_1_/S; and then followed by nocodazole treatment which disrupts microtubules required for cell mitosis to prevent cell division and arrest cells at G_2_/M. As the normal mitosis is inhibited and the cell does not divide, the DNA that replicated in the S phase cannot split between two daughter cells, resulting in doubled DNA content in the cell nucleus [32]. Further, the TEM images show the presence of larger chromatin clusters in the nucleus of the cells at G_2_/M phase, compared to that of cells at G_1_/S phase (Figure 10).

**Figure 11:**
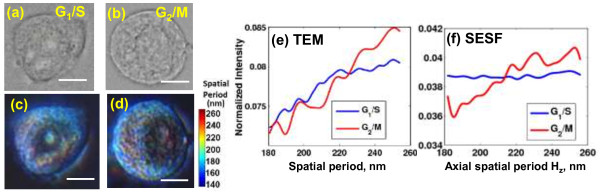
**Characterization of structural changes at different cell-cycle phases using SESF. (a,b)** The bright-field and **(c,d)** SESF images of unstained HeLa cells arrested at G_1_/S and G_2_/M phases of the cell nuclei. Scale bar: 10 μm. The color bar shows the corresponding value of spatial period in the colored images of **(c)** and **(d)**. **(e-f)** The comparison of spatial-period profile extracted from **(e)** TEM images and **(f)** SESF images.

Figure 11 show bright-field and SESF images of cells at G_1_/S and G_2_/M phase, respectively, using an objective with NA = 0.5. The changes in DNA content or chromatin cluster are not visible in the bright-field images, but the SESF images show a significantly red-shifted color in the nucleus of G_2_/M, which suggests an increased spatial period corresponding to the presence of larger structures. To further characterize the detailed spatial-period profile of cell internal structure, we compared the axial spatial-period profile of the nuclear structure quantified by SESF approach, averaged over 10–20 cells, shown in Figure 11f, with the spatial-period profile extracted from TEM images of the cell nuclei via Fourier analysis, averaged over ~10-20 cells, shown in Figure 11e at G_1_/S and G_2_/M phase, respectively [20]. They both suggest a significant increase in spatial period for cells in G_2_/M compared to those in G_1_/S. Such structural difference is clearly not detectable in bright-field microscopy.

**Figure 12:**
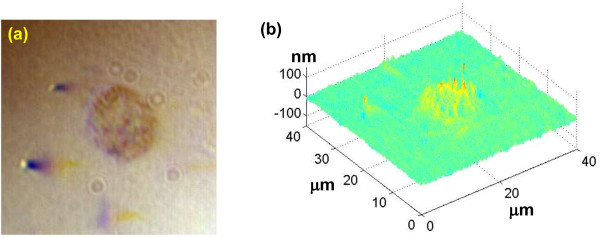
**The representative (a) bright-field image and (b) the corresponding OPD map from a cell at G**_**1**_**/S phase.**

**Figure 13:**
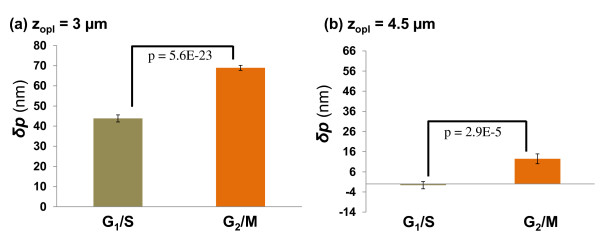
**The depth-resolved changes in OPD (δp) at two fixed optical-depth locations within the nuclei ((a) 3 and (b) 4.5 μm) for cells at G**_**1**_**/S and G**_**2**_**/M phase.** The two-sided *p*-value is shown on each figure, calculated from the student *t*-test.

The structural difference can also be detected by the OPD changes derived from the SL-QPM system. Figure 12 shows a representative OPD map at the G_1_/S phase. Figure 13 shows the averaged OPD difference δp at two different depths, by statistical analysis of ~90 nuclei at G_1_/S and G_2_/M, respectively. Overall, a significant nanoscale increase in the average OPD value is seen at both optical depths (3 and 4.5 μm) within cell nuclei at G_2_/M compared to those at G_1_/S [31]. The higher DNA content at G_2_/M phase increases the nuclear density and refractive index, resulting in a higher OPD value. Such nanoscale change in OPD as a result of the changes in DNA content is also not easily detectable in conventional microscopy.

### Nanoscale structural changes in pre-cancerous cervical cells

**Figure 14:**
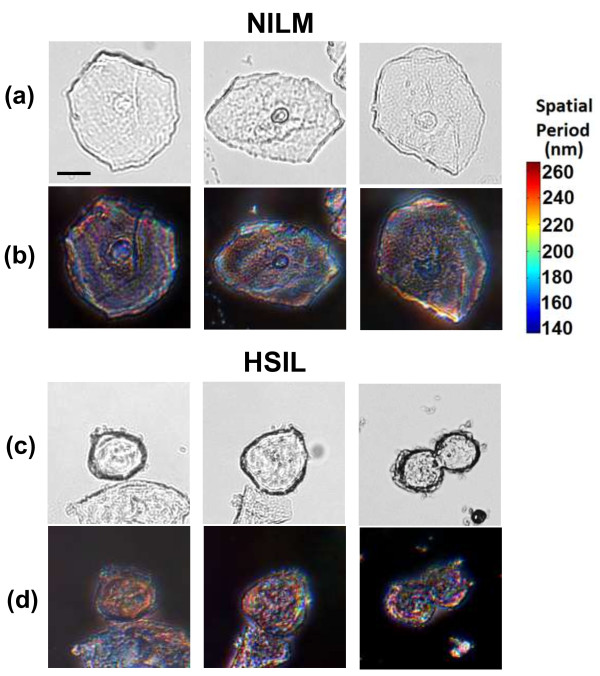
**Characterization of structural changes in pre-cancerous cervical cells using SESF.** The **(a,c)** bright-field and **(b,d)** SESF images of normal squamous epithelial cells (NILM) and high-grade squamous intraepithelial (HSIL) cells. The scale bar is 10 μm.

To evaluate the structural changes in pre-cancerous cells, we used SESF to analyze the cervical cytology specimens of 18 patients, among whom 9 diagnosed with negative intraepithelial lesion or malignancy (NILM, normal group) and 9 diagnosed with high-grade squamous intraepithelial lesion (HSIL, high-grade pre-cancerous group). Figure 14 shows the representative bright-field (a,c) and corresponding SESF images (b,d) for the unstained normal squamous cells from a patient with NILM and high-grade precancerous cells from a patient with a HSIL cytology diagnosis. The bright-field images do not reveal internal nuclear structural difference, except for the overall size difference. However, the SESF images exhibit a distinct red-shifted color in the nuclei of high-grade precancerous cells (Figure 14d), compared to those of normal cells (Figure 14b) [21]. This color shift suggests an increased spatial period in the axial intra-nuclear structure of the pre-cancerous cells, according to Eq. (11). The true color in the SESF image can be quantitatively converted into dominant wavelength based on the color-vision algorithm [21]. The dominant axial structure within the nuclei can be further quantified as the axial spatial period (Hz) derived from dominant wavelength and Eq. (11). The colorbar in Figure 14 presents the conversion between each color and dominant axial spatial period.

**Figure 15:**
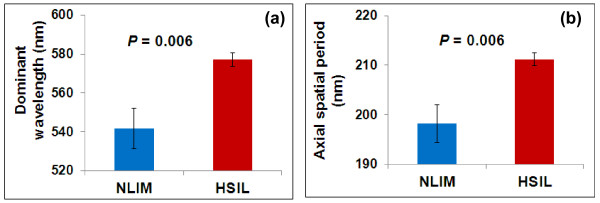
**Statistical analysis of (a) dominant wavelengths and (b) the corresponding dominant axial spatial period in the nuclei of normal squamous epithelial cells (NILM) and high-grade squamous intraepithelial (HSIL) cells.** The error bar is standard error. The p-value of the two-sided p-value of student *t*-test assuming unequal variance.

As the cell-to-cell variation is often significant, to confirm that such color shift in the cell nuclei of pre-cancerous cells is also statistically significant, we evaluated ~20 cells per patient and then took the average value of the dominant wavelengths and their corresponding average dominant axial spatial periods as a representative value for the patient. As shown in Figure 15, both the average dominant wavelength and the corresponding axial spatial period show statistically significant differences between NILM and HSIL (*p*-value = 0.006). This result suggests that the internal structures of the high-grade pre-cancerous cell nuclei exhibit an increased axial spatial period compared with those in normal cells from the NILM patients [21]. This proof-of-concept study shows the potential of SESF to detect structural changes in pre-cancerous cells not detectable with conventional light microscopy.

### Nanoscale structural changes in breast tissue

We also conducted a proof-of-concept study using archived breast tissue to investigate how nuclear nano-architecture changes in breast tumorigenesis with SL-QPM [33]. We analyzed the OPD values in the cell nucleus from well-annotated archived histology specimens processed according to standard clinical protocol. The specimens came from a total of 154 women: 24 healthy patients undergoing reduction mammoplasty; 14 patients with benign lesions; 25 patients with proliferative lesions (10 without atypia, 15 with atypia without co-existing IBC); and 32 patients with IBC whose histologically normal cells adjacent to tumor (‘malignant-adjacent’ normal) were analyzed; and 59 IBC patients whose malignant cells were analyzed. Among these lesions, normal, non-proliferative benign and proliferative lesions without atypia are considered as low-risk lesions (relative risk of 1–1.88 [34]) and patients with these lesions are not treated; while proliferative lesions with atypia has a significantly increased risk for breast cancer (relative risk of 4.24) and patients with this lesion are typically treated by both surgery and chemopreventive drug. The malignant-adjacent normal cells are no longer “normal”, because although these cells appear microscopically “normal” to pathologists, malignant tumor is already present in adjacent locations. Those malignant cells present microscopically detectable features characteristic of cancer cells. Therefore, this sequence of normal to benign, to proliferative without atypia to with atypia, to malignant-adjacent normal, to malignant cells represents a progressively increased severity in breast tumorigenesis.

**Figure 16:**
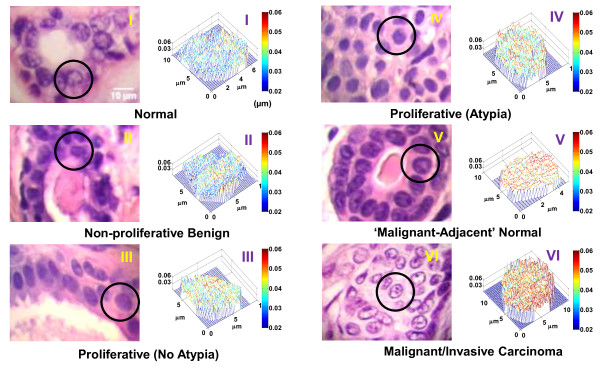
**Representative conventional images of breast biopsies and structure-derived optical pathlength difference (OPD) map from a single cell nucleus (marked in circles) from (I) normal cells from a healthy patient; (II) cells labeled as fibrocystic changes from a non-proliferative benign patient; (III) cells labeled as ductal epithelial hyperplasia from a patient with concurrent apocrine metaplasia and cystic changes; (IV) cells labeled as atypical lobular hyperplasia; (V) cells as “normal” defined by the expert pathologist from a patient with invasive breast carcinoma (‘malignant-adjacent’ normal); and (VI) cells labeled as “malignant” from a patient with invasive breast carcinoma.** The color bar represents the OPD value from the cell nucleus.

We first constructed the OPD map at the optical depth of interest for the cell nuclei and Figure 16 shows representative pseudo-color OPD maps from cell nuclei for each of the 6 categories (I to VI in Figure 16). The color and spatial distribution in these OPD maps reveal a progressive change from normal through malignant, which correlates to breast tumorigenesis. The OPD maps from low-risk lesions (normal, benign, proliferative without atypia) show similar pattern, while the high-risk lesion and malignant lesions are distinctly different. Most importantly, the OPD maps from malignant-adjacent “normal” cells, although histologically normal-appearing, exhibit a great similarity to those of malignant cells, indicative of cancer-like nano-morphology signatures in these malignant-adjacent “normal” cells.

**Figure 17:**
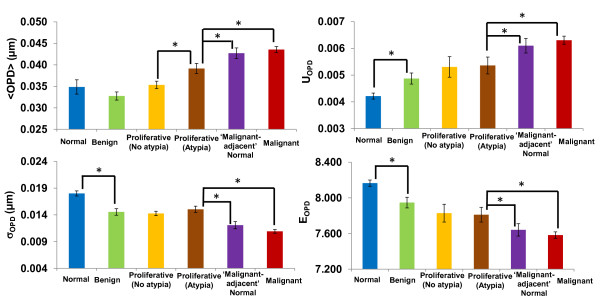
**Statistical analysis of nano-morphology markers in annotated breast tissue with different pathology types.** The * sign indicates statistical significance between two groups with two-sided *p*-value < 0.05.

To quantify the nano-morphology signatures reflected in these OPD maps, we extracted four quantitative markers from the OPD map of each cell nucleus, including average nuclear OPD < OPD>, intra-nuclear standard deviation of OPD σ_OPD_, entropy E_OPD_ and uniformity U_OPD_. Their mean value of 40–60 cell nuclei is used as the characteristic marker for each patient. As we previously shown in the cell cycle experiment, the < OPD > is associated with nuclear density; and intra-nuclear σ_OPD_, E_OPD_, and U_OPD_ are different measures of structural heterogeneity from each cell nucleus. As shown in Figure 17, these four nano-morphology markers progressively changed in parallel with the development of breast tumorigenesis. In particular, <OPD > values in low-risk lesions are similar without statistical significance (ANOVA, P > 0.05). On the other hand, <OPD > is significantly increased in high-risk lesion (proliferative with atypia), and such change in even more elevated in patients with malignancy. The structural heterogeneity measures (σ_OPD_, E_OPD_ and U_OPD_) show a distinct difference between normal and benign lesions, suggesting that they are also sensitive to abnormal changes in the breast tissue, even for low-risk benign conditions. Their values are significantly different from patients with malignancy. Such nano-morphology changes cannot be detected by quantitative measure of microscopic features of the nucleus. For example, the nuclear size, which is the most important measure in diagnosing breast cancer, does not show any statistical significance between normal and patients with malignant-adjacent normal cells [33]. The only significant change is seen between malignant cells and the rest of non-malignant cells (including normal, benign, proliferative lesions and ‘malignant-adjacent’ normal cells), in agreement with conventional pathology.

This proof-of-concept study demonstrates that nuclear nano-morphology markers, derived from SL-QPM, show great promise to detect breast cancer with a high accuracy beyond conventional pathology. These nuclear nano-morphology markers are based on the detection of nanoscale structural characteristics, which otherwise cannot be appreciated using light microscopy or digital image analysis.

## Conclusions

The analysis of nanoscale structural characteristics has shown some promise in detecting cancer before the microscopically visible changes become evident and several proof-of-concept studies using clinical patient samples have also shown its feasibility as an earlier or more sensitive marker for cancer detection or diagnosis in a clinical setting. From a basic science perspective, we will need further understanding and validation to identify specific 3D nanoscale structural characteristics as a downstream manifestation of complex molecular changes in carcinogenesis via direct super-resolution imaging of the 3D nuclear architecture in carcinogenesis using well-annotated human tissue from different tumor types. It remains an important biophysical question to be addressed. For example, what are specific 3D high-order chromatin structural alterations at the nanoscale in the development of cancer? How are they affected by molecular heterogeneity of the tumor? How do they change as a response to anti-cancer treatment? Ultimately, the elucidation of specific nanoscale structural characteristics together with the development of cost-effective and clinically applicable tools to accurately interrogate these nanoscale structural features in a high-throughput manner will have a significant potential clinical impact in bringing a new class of cancer markers for “personalized” cancer detection, diagnosis, prognosis, or monitoring of drug response or tumor recurrence.

### Ethics statement

All research was performed with the approval of the institutional review board at University of Pittsburgh.

## Competing interests

The authors (YL, SU) declare competing financial interests. The SL-QPM system was filed for patent application, which has been licensed to a private company.

## Authors' contributions

SU, SA and RB performed the experiments and analyzed the data. YL, SU and SA wrote the paper. YL supervised the project. All authors read and approved the final manuscript.
